# Efficient Differentiation of Mouse Embryonic Stem Cells into Insulin-Producing Cells

**DOI:** 10.1155/2012/201295

**Published:** 2012-08-05

**Authors:** Szu-Hsiu Liu, Lain-Tze Lee

**Affiliations:** Pharmacognosy Lab, Herbal Medicinal Product Technology Division, Industrial Technology Research Institute, Hsinchu 30011, Taiwan

## Abstract

Embryonic stem (ES) cells are a potential source of a variety of differentiated cells for cell therapy, drug discovery, and toxicology screening. Here, we present an efficacy strategy for the differentiation of mouse ES cells into insulin-producing cells (IPCs) by a two-step differentiation protocol comprising of (i) the formation of definitive endoderm in monolayer culture by activin A, and (ii) this monolayer endoderm being induced to differentiate into IPCs by nicotinamide, insulin, and laminin. Differentiated cells can be obtained within approximately 7 days. The differentiation IPCs combined application of RT-PCR, ELISA, and immunofluorescence to characterize phenotypic and functional properties. In our study, we demonstrated that IPCs produced pancreatic transcription factors, endocrine progenitor marker, definitive endoderm, pancreatic **β**-cell markers, and Langerhans **α** and **δ** cells. The IPCs released insulin in a manner that was dose dependent upon the amount of glucose added. These techniques may be able to be applied to human ES cells, which would have very important ramifications for treating human disease.

## 1. Introduction

Human and mouse embryonic stem (ES) cells are capable of spontaneous differentiation into insulin-producing cells, among many other cell types. ES cells can be induced to preferentially differentiate into insulin-producing cells (IPCs) by changing the composition of the culture medium and causing expression of dominant transcription factor genes which are involved in pancreas development [1, 2]. In previous studies, there are two main strategies for the differentiation of ES cells into IPCs: (i) embryoid body formation and (ii) definitive endoderm formation [3–5]. Because after spontaneous differentiation the number of specifically differentiated cell types is relatively low, the application of defined differentiation factors and selection of lineage-specific progenitor cells seems to be necessary for directed differentiation of ES cells into the desired cell types [6, 7]. Differentiated cells can be obtained within approximately 33 days. 

Until now, there is no report to directly induce definitive endoderm and pancreatic cells in monolayer cells at the same time. Here, we present a strategy for the differentiation of ES cells into IPCs by a two-step differentiation protocol comprising of (i) the formation of definitive endoderm in monolayer culture by activin A, and (ii) this monolayer endoderm being induced to differentiate into IPCs by nicotinamide, insulin, and laminin. The small bioorganic molecules can control cellular processes by modulation of metabolism, signal transduction pathways and gene regulation [8–18]. In our study, we demonstrated that bioorganic molecules provide key information to modulation of stem cell proliferation and differentiation at 7 days. We also combined application of the three analytical methods presented here—RT-PCR, ELISA, and immunofluorescence to characterize phenotypic and functional properties.

## 2. Materials and Methods

### 2.1. Chemicals

Leukemia inhibitory factor (LIF) was purchased from Chemicon. Mouse gelatin was purchased from BD (Becton, Dickinson and Company). Culture media and fetal bovine serum (FBS) were purchased from Hyclone Laboratories Inc. Activin A was purchased from R&D system. Other chemicals were purchased from Sigma-Aldrich.

### 2.2. Cell Culture and Differentiation

Undifferentiated ES-D3 murine embryonic stem cell lines (BCRC, 60205) were cultured on a feeder layer of mouse embryonic fibroblasts on gelatin-coated flasks in Dulbecco's modified Eagle's medium (DMEM) with 4 mM L-glutamine adjusted to contain 1.5 g/L sodium bicarbonate and 4.5 g/L glucose, 0.1 mM 2-mercaptoethanol supplemented with 15% fetal bovine serum (FBS), 1400 units/mL leukemia inhibitory factor (LIF) at 37°C and 5% CO_2_. Subsequently, ES-D3 cells were transferred onto gelatin-coated flasks for 30 min to remove the feeder layer. ES-D3 cells were seeded at 1 × 10^6^ cells per well to collagen-I-coated plates in DMEM/F-12 medium supplemented with 2 mM L-glutamine, 100 *μ*M nonessential amino acids, 10 ng/mL activin A, 10 mM nicotinamide, and 1 *μ*g/mL laminin with 10% FBS overnight. ES-D3 cells were next exposed to DMEM/F-12 medium supplemented with 2 mM L-glutamine, 100 *μ*M nonessential amino acids, 10 ng/mL activin A, 10 mM nicotinamide, 25 *μ*g/mL insulin, and 1 *μ*g/mL laminin with 2% FBS for 6 days.

### 2.3. RNA Isolated and RT-PCR Analysis

Total RNA was isolated using PureLink Micro-to-Midi Total RNA (Invitrogen), according to the manufacturer's recommended protocol. RNA samples (1 *μ*g/reaction) were reverse-transcribed with Superscript (Invitrogen) in the presence of oligo-dT, and the RT reaction was used for amplification with *Taq *polymerase. The resulting cDNA was amplified using specific primers. The sequences were as follows: definitive endoderm marker Sox7 (forward 5′-CCA TAG CAG AGC TCG GGG TC-3′; reverse 5′-GTG CGG AGA CAT CAG CGG AG-3′), endocrine progenitor marker Ngn3 (forward 5′-TGG CGC CTC ATC CCT TGG ATG-3′; reverse 5′-AGT CAC CCA CTT CTG CTT CG-3′), pancreatic transcription factors Pax4 (forward 5′-ACC AGA GCT TGC ACT GGA CT-3′; reverse 5′-CCC ATT TCA GCT TCT CTT GC-3′), pancreatic transcription factors Pax6 (forward 5′-TCA CAG CGG AGT GAA TCA G-3′; reverse 5′-CCC AAG CAA AGA TGG AAG-3′), pancreatic *β*-cell markers Insulin 1 (forward 5′-TAG TGA CCA GCT ATA ATC AGA GAC-3′; reverse 5′-CGC CAA GGT CTG AAG GTC-3′), pancreatic *β*-cell markers Insulin 2(forward 5′-CCC TGC TGG CCC TGC TCT T-3′; reverse 5′-AGG TCT GAA GGT CAC CTG CT-3′), Langerhans *α*- cells Glucagon (forward 5′-CAT TCA CAG GGC ACA TTC ACC-3′; reverse 5′-CCA GCC CAA GCA ATG AAT TCC-3′), Amylase (forward 5′-CAG GCA ATC CTG CAG GAA CAA-3′; reverse 5′-CAC TTG CGG ATA ACT GTG CCA-3′).

Langerhans *δ*-cells Somatostatin (forward 5′-TCG CTG CTG CCT GAG GAC CT-3′; reverse 5′-GCC AAG AAG TAC TTG GCC AGT TC-3′), *β*5-tubulin (forward 5′-TCA CTG TGC CTG AAC TTA CC-3′; reverse 5′-GGA ACA TAG CCG TAA ACT GC-3′). For amplification, an initial reverse transcription step was followed by denaturing step (94°C for 5 minutes) and then by 30 cycles of denaturing (94°C for 30 seconds), annealing (60°C for 30 seconds), and extending (72°C for 30 seconds), followed by 7 minutes at 72°C for elongation. Glucagon and Insulin 2 annealing conditions are 55°C for 30 seconds, 65°C for 30 seconds, respectively. The PCR products produced were separated by electrophoresis on 2% agarose gel.

### 2.4. Immunofluorescence

The cells were fixed for 30 minutes at room temperature in 4% paraformaldehyde, then washed three times in PBS. The cells were blocked for 30 minutes in PBS plus 0.2% Triton X-100, 1% bovine serum albumin (BSA). Anti-C-peptide primary antibody (Cell Signaling Technology, Danvers, MA) was diluted 1 : 500 in PBS and incubated for 60 minutes at 37°C. The cells were rinsed three times with PBS and then incubated with fluorescence-labeled specific secondary antibody diluted in PBS with 0.5% BSA at 37°C for 45 minutes. After washing, cells were incubated with DAPI at dilution 1 : 1000 in PBS for 10 minutes.

### 2.5. Insulin Content

Differentiated cells were seeded at 1 × 10^6^ cells per well in a 24 well culture plate and incubated overnight in culture media and were grown for 24 hours in DMEM/F-12 medium without insulin. Cells were then washed twice and preincubated at 37°C for 1 hour with Krebs-Ringer bicarbonate HEPES buffer (KRBH) containing 2.5 mM glucose. Cells were then incubated for 1.5 hours in KRBH buffer (contain 50 *μ*M tolbutamide) with 2.5, 5.5, and 12.5 mM glucose. Insulin content was determined by ELISA (Mercodia).

### 2.6. Statistical Analysis

All data were performed in triplicate, and all experiments were repeated at least three times. Data were presented as mean ± standard deviation (SD) and analyzed using one-way analysis of variance (ANOVA, SAS 9.1.3, USA), followed by a Tukey's test to determine any significant differences. *P* values of less than 0.05 were considered statistically significant.

## 3. Results

### 3.1. Gene Expression and Immunofluorescence Analysis of Insulin-Producing Cells

**Figure 1:**
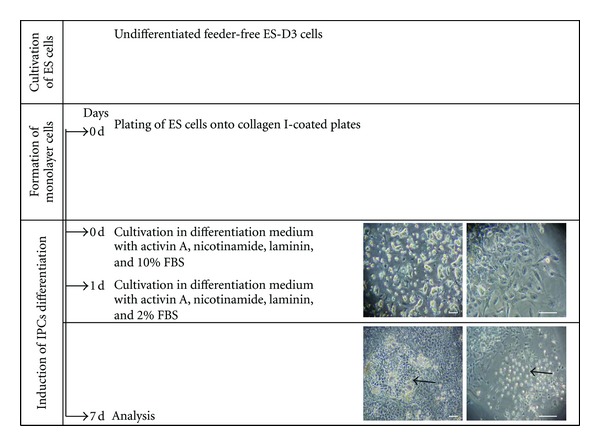
Schematic representations of the differentiation protocol from ES cells into insulin-producing cells. Undifferentiated feeder-free ES-D3 cells were cultured in collagen-I-coated plates and incubated in differentiation DMEM/F-12 medium supplemented with 2 mM L-glutamine, 100 *μ*M nonessential amino acids, 10 ng/mL activin A, 10 mM nicotinamide, and 1 *μ*g/mL laminin with 10% FBS overnight. ES-D3 cells were next exposed to DMEM/F-12 medium supplemented with 2 mM L-glutamine, 100 *μ*M nonessential amino acids, 10 ng/mL activin A, 10 mM nicotinamide, 25 *μ*g/mL insulin, and 1 *μ*g/mL laminin with 2% FBS for 6 days. Differentiated ES-D3 cells formation of islet-like clusters at 7 days. Bar = 50 *μ*m.

In preliminary experiments, we found that 10 ng/mL activin A, 10 mM nicotinamide, 25 *μ*g/mL insulin, and 1 *μ*g/mL laminin under low serum are an ideal condition for differentiation of monolayer endoderm cells into IPCs at 7 days (shown in Figure 1).

**Figure 2:**
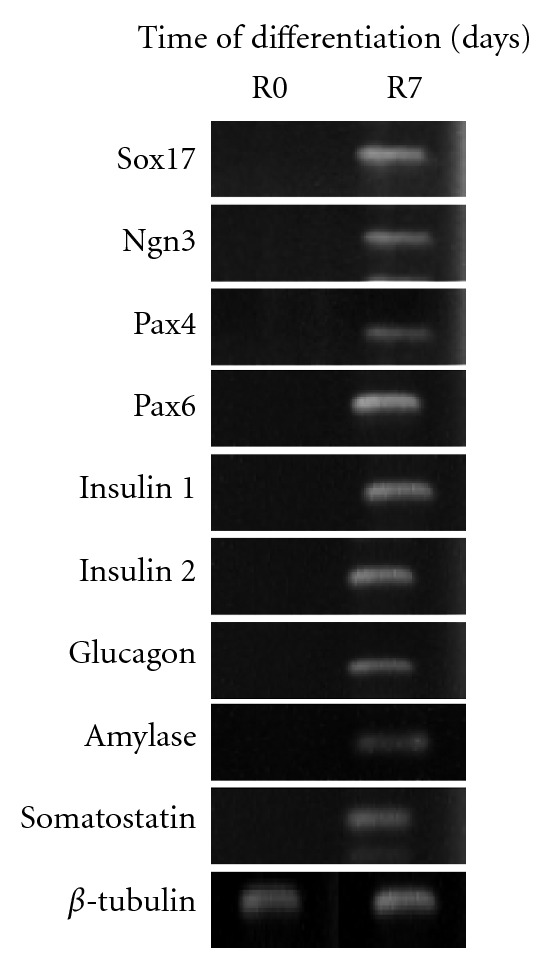
RT-PCR analysis of pancreatic-specific genes expression. RT-PCR analysis of undifferentiated R0 embryonic stem (ES-D3) cells and R7 cells (insulin-producing cells) at differentiation stages of 7 days. The *β*5-tubulin gene was used as a housekeeping-gene standard.

**Figure 3:**
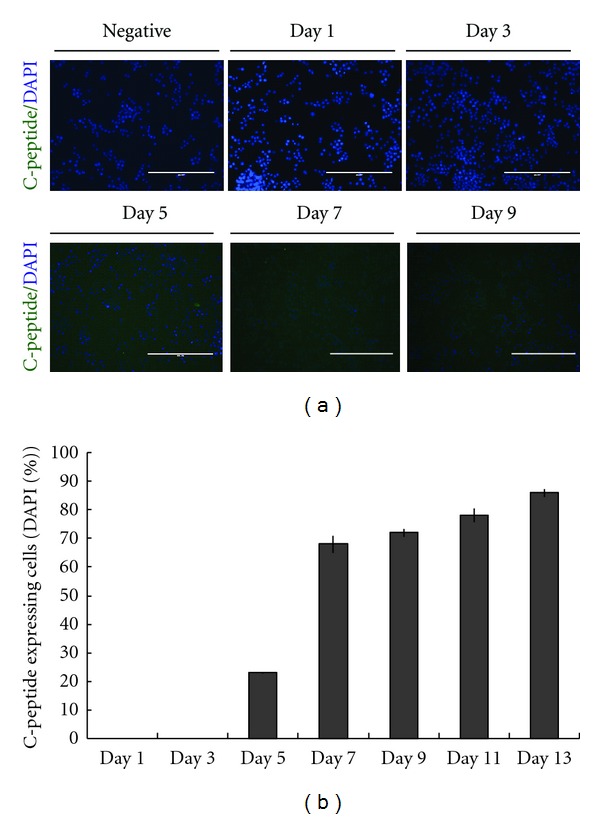
Immunofluorescence analysis of the C-peptide expressing cells. (a) Insulin-producing cells stain positive for C-peptide expression (C-peptide stain, green; DAPI stain, blue). RAW 264.7 (Mouse leukaemic monocyte macrophage cell line) is the negative control. Scale bars = 200 *μ*m. (b) Experimental time course for the differentiation of ES-D3 cells to IPCs. For the quantification of IPCs, at least 10 images for each treatment were taken using an EVOS fluorescent microscope (USA). Total cell number was quantified based on DAPI nuclear staining and C-peptide expressing cells were quantified using Image J software (NIH, US). Bars represent means ± SD from three independent experiments. *P < 0.05 significantly different from 7 days.

To assess IPCs developmental changes resulting from specific modifications of culture conditions, we evaluated the expression of various genes by a semiquantitative reverse transcription polymerase chain reaction (RT-PCR) assay. As shown in Figure 2, the results indicated that differentiated mouse ES cells expressed pancreatic transcription factors (Pax4 and Pax6), endocrine progenitor marker (Ngn3), definitive endoderm (Sox7), exocrine pancreas marker (Amylase), pancreatic *β*-cell markers (Insulin 1 and Insulin 2), and Langerhans *α*- and *δ*-cells (Glucagon and Somatostatin). Fluorescence micrographs also demonstrated pancreatic hormone C-peptide expressing in IPCs (shown in Figure 3(a)). After 7-day treatment with 10 ng/mL activin A, 10 mM nicotinamide, 25 *μ*g/mL insulin, and 1 *μ*g/mL laminin, the percentage of C-peptide expressing cells increased to 67.3 ± 2.9% (Figure 3(b)). In the time course of the next 6 days, the efficiency of C-peptide expressing does not significantly change.

### 3.2. Insulin Content and In Vitro Glucose-Stimulated Insulin Secretion

**Figure 4:**
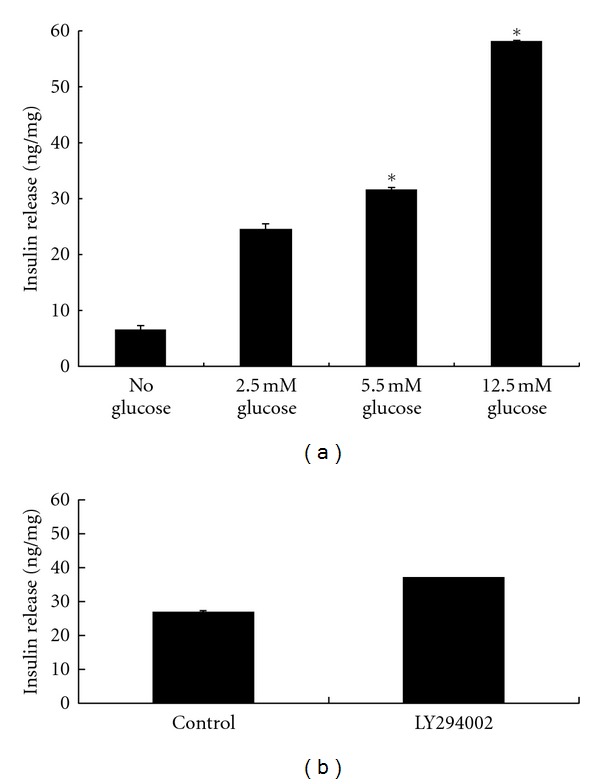
Intracellular insulin content. Insulin secretion was measured by ELISA and normalized to total cellular protein. (a) Insulin-producing cells were incubated with KRBH buffer containing glucose and 50 *μ*M tolbutamide for 1.5 hours. Bars represent means ± SD from three independent experiments (*n* = 3). *P < 0.05 significantly different from glucose. (b) Insulin-producing cells were incubated with 3 *μ*M LY294002 or not (control) for 2 hours. Bars represent means ± SD from three independent experiments (*n* = 3). *P < 0.05 significantly different from control.

ES-3D cells were treated with glucose to evaluate whether IPCs released insulin in a manner that was dose-dependent upon the amount of glucose added. As shown in Figure 4(a), it can be seen that insulin is released in a manner that is dependent directly upon the amount of glucose added. At 12.5 mM glucose, the insulin released was double the amount released at .5 mM glucose, and the amount of insulin released in cells treated with 12.5 mM glucose was approximately twice that in cells treated with 2.5 mM glucose (Figure 4(a)).

 In the previous studies insulin content was increased following mouse ES cells differentiation in the presence of the phosphatidylinositol 3-kinase inhibitor LY-294002. The results showed that insulin content was increased approximately 1.3-fold compared with untreated control (Figure 4(b)).

## 4. Conclusions

Embryonic stem (ES) cells are a potential source for insulin-producing cells (IPCs), but existing differentiation protocols are of limited efficiency. The aim has been to develop an efficient differentiation protocol, in which we could induce differentiation by small specific molecules, including activin A, laminin, nicotinamide, and insulin. Permeable small molecules can control cellular processes by modulating signal transduction pathways, gene expression, or metabolism and have been effectively used in ESC differentiation protocols. These molecules, alone or in combination with specific growth factors and hormones, will likely provide key information to design specific culture media in order to obtain IPCs [8–18]. Until now, there is no report to directly induce definitive endoderm and pancreatic cells at the same time by small specific molecules.

We here present a strategy for the differentiation of mouse ES cells into IPCs by a two-step differentiation protocol comprising of (i) the formation of definitive endoderm in monolayer culture by activin A, and (ii) this monolayer endoderm being induced to differentiate into IPCs by nicotinamide, insulin, and laminin. Activin A, a member of the transforming growth factor-*β* (TGF-*β*) superfamily, has been shown to induce endodermal differentiation of the cells of this endodermal monolayer under low serum conditions. In most of the recent studies, activin A starting from the beginning of *in vitro *differentiation monolayer can be added to cause human ES cells to differentiate into definitive endodermal cells [6]. Nicotinamide (also known as niacinamide) is a form of vitamin B3, which enhances the* in vitro* differentiation of cultured human pancreatic cells, favoring the expression of insulin, glucagon, and somatostatin [14–16]. Laminin enhances IPCs differentiation with increases in insulin and Glut2 gene expressions, proinsulin, and insulin release in response to elevated glucose concentration [17, 18].

In this study, pancreatic *β*-cells (Insulin 1 and Insulin 2) and Langerhans cells' markers (Glucagon and Somatostatin) could be identified in differentiated ES cells. Fluorescence micrographs also demonstrated that pancreatic hormone C-peptide was expressed in IPCs, the percentage of C-peptide expressing cells about 67.3 ± 2.9%. Insulin content was increased in a glucose-dependent manner. After treatment with phosphatidylinositol 3-kinase inhibitor LY-294002, insulin was increased to 1.3-fold of that in untreated cells. In summary, these studies show that small molecules induce definitive endoderm and pancreatic cells in monolayer cells at the same time in the differentiation process. Our differentiation system represents an efficient protocol to direct mouse ES cells into the pancreatic lineage by generating IPCs and is applicable to further strategies for the improvement of *in vitro *differentiation into functional insulin-producing cells.
